# Wilforine inhibits rheumatoid arthritis pathology through the Wnt11/β-catenin signaling pathway axis

**DOI:** 10.1186/s13075-023-03224-2

**Published:** 2023-12-14

**Authors:** Yurong Huang, Yanhui Peng, Hui Li, Chen Li, Yajie Wu, Xiaomei Wang, Jun Chang, Chenggui Miao

**Affiliations:** 1 Department of Pharmacology, School of Integrated Chinese and Western Medicine, Anhui University of Chinese Medicine, Hefei, 230012 Anhui Province China; 2 Department of Humanistic Nursing, School of Nursing, Anhui University of Chinese Medicine, Hefei, China; 3 Department of Orthopaedics, the First Affiliated Hospital, Anhui Medical University, Hefei, 230032 China; 4 Anhui Public Health Clinical Center, Hefei, China

**Keywords:** Rheumatoid arthritis, Fibroblast-like synoviocytes, Wilforine, *Tripterygium wilfordii* Hook. f., Wnt11/β-catenin signaling pathway

## Abstract

**Background:**

Wilforine (WFR) is a monomeric compound of the anti-RA plant *Tripterygium wilfordii* Hook. f. (TwHF). Whether WFR has anti-RA effect, its molecular mechanism has not been elucidated.

**Aim of the study:**

Our study aims to clarify how WFR inhibits fibroblast-like synovial cells (FLS) activation and improves RA through Wnt11 action on the Wnt11/β-catenin signaling pathway.

**Methods:**

The therapeutic effect of WFR on collagen-induced arthritis (CIA) rats was evaluated using methods such as rat arthritis score. The inhibitory effects and signaling pathways of WFR on the proliferation and inflammatory response of CIA FLS and RA FLS were studied using ELISA, CCK-8, RT-qPCR, Western blot, and immunofluorescence methods.

**Results:**

WFR could effectively alleviate the arthritis symptoms of CIA rats; reduce the levels of IL-6, IL-1β, and TNF-α in the peripheral blood of CIA rats; and inhibit the expression of MMP3 and fibronectin. The data showed that WFR has a significant inhibitory effect on FLS proliferation. Furthermore, WFR inhibited the activation of Wnt/β-catenin signaling pathway and decreased the expression of Wnt11, β-catenin, CCND1, GSK-3β, and c-Myc, while the effects of WFR were reversed after overexpression of Wnt11.

**Conclusions:**

WFR improves RA by inhibiting the Wnt11/β-catenin signaling pathway, and Wnt11 is the direct target of WFR. This study provides a new molecular mechanism for WFR to improve RA and contributes to the clinical promotion of WFR.

**Supplementary Information:**

The online version contains supplementary material available at 10.1186/s13075-023-03224-2.

## Introduction

Rheumatoid arthritis (RA) involves multiple joint injuries on both sides and can lead to cartilage and bone damage and disability. Systemic inflammatory processes may involve other tissues and organs [1]. Inadequately treated active RA can lead to joint disability and other complications that reduce the quality of life [2]. Clinically, early RA presents fatigue, joint swelling and pain, and morning stiffness, while undertreated RA presents complex clinical symptoms. RA can occur at any age, but most occur between the ages of 20 and 55. For most RA patients, early treatment can slow disease progression and irreparable joint damage [3].

The primary effector cells in the pathophysiology of RA are fibroblast-like synoviocytes (FLS) [4]. Abnormal proliferation of FLS can secrete IL-6, IL-1β, and TNF-α and stimulate the inflammatory response of macrophages and T cells [5]. Activated FLS also recruit more inflammatory cells into synovial tissue by secreting chemokines [6]. The interaction between FLS and inflammatory immune cells plays a significant role in the pathogenesis of RA.

*Tripterygium wilfordii* Hook. f. (TwHF) is an effective and key medicinal plant for the clinical treatment of RA in China [7]. TwHF extract has statistically significant and clinically significant improvements in RA symptoms and has acceptable safety. Clinical trials have shown that TwHF monotherapy is not inferior to methotrexate (MTX) monotherapy, while the combined treatment of MTX and TwHF is more effective in controlling disease activity in active RA patients than MTX monotherapy [8]. Compared to MTX monotherapy, the combination of TwHF and MTX in the treatment of RA may be a better strategy. Research has shown that the optimal combination of TwHF+MTX treatment may be 30–60 mg/day of TwHF and MTX (~10 mg/week) [9]. Therefore, TwHF is a potential source of novel anti-RA monomer compounds.

Wilforine (WFR) is one of the monomeric components of TwHF and can be used as a quality marker for the medicinal herb [10, 11]. Given the significant anti-RA effects of TwHF, it is worth noting whether its monomer WFR has a good anti-RA effect.

Wnts are exocrine glycoproteins whose signal transduction involves multiple genes and receptors. Activation of the Wnt/β-catenin pathway can trigger the production of MMPs and other proteases, leading to the breakdown of the proteoglycan matrix and disrupting the balance of extracellular matrix (ECM) degradation [12]. Glycogen synthase kinase-3β (GSK-3β), a serine/threonine protein kinase, has been implicated as a regulator of the inflammatory response. Studies have shown that the inhibition of GSK-3β can be used as an effective therapeutic agent, and serum levels of IL-1β, IL-6, TNF-α, and IFN-γ in CIA mice were also significantly decreased in dose-dependent manners by treatment with GSK-3β inhibitors [13]. In addition, serum levels of MMP3 reflect positively RA disease activity, joint and bone injury, and radiological erosion and predict disease outcome and drug responsiveness [14]. Wnt11 is one of the extracellular Wnt proteins involved in the Wnt signaling pathway. Wnt11 can activate the Wnt signaling by inducing the recombinant RSPO2/leucine-rich repeat containing G protein-coupled receptor 5 (LGR5) complex through the noncanonical Wnt pathway [15]. Wnt11 also promotes angiogenesis by activating the atypical Wnt11/PKC/JNK signaling pathway [16]. The Wnt11-FZD7-DAAM1 signal supports tumor initiation ability and melanoma amoeba-like invasion [17]. The lack of Wnt11 alone does not significantly regulate the body’s hypertrophy response to pressure overload. Wnt11 may need to collaborate with other noncanonical Wnt signaling pathway to regulate stress-induced hypertrophy response [18]. Under pressure overload, heart Wnt5a and Wnt11 promote tissue fibrosis through the crosstalk of FZD5 and epidermal growth factor receptor (EGFR) signals [19].

Given that synovial hyperplasia and angiogenesis during RA have certain similarities with cardiac tissue hyperplasia and tumor pathology, we focus on the expression changes and roles of Wnt11 in RA. Pre-experiments showed a significant increase in Wnt11 expression in synovial tissue of RA patients and RA model CIA rats. The monomer component WFR in TwHF significantly improved symptoms in CIA rats and inhibited the Wnt11 expression. Based on this, we further explore whether Wnt11 promotes RA by influencing the Wnt signaling pathway and whether WFR inhibits RA pathology through the Wnt11/β-catenin signaling pathway. This work will confirm that Wnt11 is a new diagnostic and therapeutic target for RA and also elucidate the mechanism by which WFR improves RA, providing scientific basis for its clinical application.

## Materials and methods

### Animal and patient samples

CIA rats were prepared using SPF grade SD rats, male, weighing 160–180 g (production license no. SCXK (Liao) 2020-0001; Experimental Animal Ethics Certificate No.: AHUCM-rats-2022110; February 7, 2022). Mixed bovine type 2 collagen and complete Freund’s adjuvant CFA in a 1:1 ratio to prepare a modeling reagent. First immunization is as follows: used bovine type 2 collagen as an emulsifier, thoroughly mixed with complete Freund’s adjuvant for 30 min, and injected 100 μL of modeling reagent into the tail root (avoiding blood vessels) and subcutaneous area of the back of rats, respectively. Secondary immunization is as follows: used incomplete Freund’s adjuvant as an emulsifier for bovine type 2 collagen and performed secondary immunization on the twenty-first day after the first immunization. The method and injection amount were the same as the first immunization.

Medical ethics was approved by the First Affiliated Hospital of Anhui Medical University, and the approval document no. is PJ-YX2021-026. RA joint synovial samples and healthy control samples were obtained from RA patients and non-RA patients in the orthopedics department of the hospital. Patients agree and sign informed consent forms.

### FLS cultivation

FLS were isolated and cultured using tissue block primary cell culture method. FLS cells were obtained by collagenic digestion, and the synovium tissue was rinsed on ice five times with PBS containing 1% penicillin and streptomycin. Then cut the synovial tissue into 1 mm^3^ tissue blocks, transferred the tissue blocks into cell culture flasks, added high sugar DMEM medium (Beyotime, China) containing 10% bovine serum (Beyotime, China), and cultured the FLS in a 37 °C, 5% CO_2_ incubator. The CIA FLS used in this work were the 3rd~6th generation, and human RA FLS were purchased from Saibaikang Biotechnology Co., Ltd. (Shanghai, China).

### Drug treatment methods

In *in vivo* studies, the positive drug was methotrexate (MTX) (Shanghai SINE Pharmaceutical Co., Ltd., Lot No. 210502). Based on the drug dose and human-rat body surface area exchange algorithm, the MTX gavage dose of CIA rats was 0.75 mg/kg. WFR (wilforine) (molecular formula: C43H49NO18, case no.: 11088-09-8), purity ≥ 98%, was purchased from Deruike Biotechnology Co., Ltd. (Chengdu, China). For the intragastric dose of WFR in rats, based on the human-rat body surface area exchange algorithm and referred to the experimental dose of triptolide, the reasonable experimental dose range of WFR in rats is 8–56 μg/kg. According to the literatures [7–11], the reasonable experimental dose range for WFR on FLS is 100–700 nM.

According to the WFR dose screening results, the three doses of in vivo experiments used in this study were 40 μg/kg (low dose), 48 μg/kg (medium dose), and 56 μg/kg (high dose). For in vivo experiments in rats, we used water as a solvent to suspend and dissolve WFR, and added 10% medicinal starch to increase viscosity, with the aim of fully mixing WFR. Rats were divided into normal group, model group (CIA), model+low-dose group (CIA+low dose), model+medium-dose group (CIA+medium dose), model+high-dose group (CIA+high dose), and negative control group (NC group, medicinal starch group), with 10 rats in each group.

The three doses for WFR cell experiments were 200 nM (low dose), 300 nM (medium dose), and 400 nM (high dose). For FLS in vitro experiments, we used DMSO to dissolve WFR. FLS were divided into normal group, model group (CIA FLS), model+low-dose group (CIA+low dose), model+medium-dose group (CIA+medium dose), model+high-dose group (CIA+high dose), and NC group (DMSO group).

### Scoring of arthritis

The specific criteria of the arthritis scores were listed as follows: grade 0, no redness and swelling, marked as 0 points; grade 1, redness and swelling of little toe joints, marked as 1 point; grade 2, redness and swelling of all joints and toes, denoted as 2 points; grade 3, redness and swelling below the ankle joint, labeled as 3 points; and grade 4, redness and swelling of all joints including ankle joints, recorded as 4 points. The cumulative score of each joint was the arthritis index of each rat [20].

### Histopathological examination

The knee joints of rats were fixed in 4% paraformaldehyde for 24 h and demineralized in 10% ethylene diamine tetraacetic acid solution. Subsequent to dehydration with gradient alcohol, clarification, and paraffin embedding, the samples were sectioned consecutively. After the sections were stained, dehydrated, transparent, and sealed, the histopathological changes and severity were observed under microscope (CX41, Olympus, Japan).

### Cell counting Kit-8 (CCK-8)

It planted FLS in a 96-well plate, waited for FLS to grow to 80% of the bottom area of the well, and then started adding drugs for testing. The effects of different doses of WFR on the proliferation of FLS were detected according to the instructions of the CCK-8 kit (Biosharp, China).

### ELISA

The levels of IL-1β, IL-6, and TNF-α were determined by ELISA (ColorfulGene, Wuhan, China. Lot numbers: JYM0419RA, JYM0646RA, JMY0635RA). The standard solution was diluted in proper proportion to establish the standard curve. Set blank and sample holes, add sample, and add enzyme-labeled reagent except blank well. Incubated, washed, added developer solution, reacted in the dark, and then added the stop solution. The OD value was detected using an enzyme-labeled instrument (Waltham, MA, USA).

### RT-qPCR

RNA was extracted from cells by TRIzol Reagent (Invitrogen, USA) and transcribed into cDNA using reverse transcription kit (Biosharp, China). RT-qPCR was performed by SYBR RT-qPCR kit (Biosharp, China), and the results were analyzed by 2^−ΔΔCt^ method (primers were listed in Table 1).

**Table 1:** Sequences of primers for RT-qPCR

Gene	Forward primer	Reverse primer
Rat sequence		
β-catenin	ACAAGCCACAGGACTACAAGAAACG	TCAGCAGTCTCATTCCAAGCCATTG
c-Myc	AGCAGCGACTCTGAAGAAGAACAAG	GGATGACCCTGACTCGGACCTC
CCND1	GAGGCGGATGAGAACAAGCAGATC	GGAGGGTGGGTTGGAAATGAACTTC
MMP3	CGTCGGTGGCTTCAGTACCTTTC	TCACCTCCTCCCAGACCTTCAAAG
Fibronectin	AGGCACAAGGTCCGAGAAGAGG	GGTCAAAGCATGAGTCATCCGTAGG
β-actin	CAACTGGCGGGACTTTCTCAAGG	CAGCACTGTGTTGGCATAGAGGTC
GSK-3β	CAATCGCACTGTGTCGCCGTCTC	GGTGTGTCTCGCCCATTTGGTAG
Wnt11	CAGGATCCCAAGCCAATAAA	GACAGGTAGCGGGTCTTGAG
Human sequence		
β-catenin	GGTGTCCAACACAGATCTGA	CCACTAGACCACTCATCTAC
c-Myc	TTCCCCTACCCTCTCAACGAC	TTCTTCCTCATCTTCTTGTTCCTCC
CCND1	CTGTGCATCTACACCGACAACTA	AGGTTCCACTTGAGCTTGTTCAC
MMP3	TCCCAGGAAGATAGCTGAGG	CAACTGCGAAGATCCACTGA
Fibronectin	AAGCTGCTGGAGCTGATAAGA	GTTACAGCCCAAACGACTGAC
β-actin	TGTCACCAACTGGGACGATA	GGGGTGTTGAAGGTCTCAAA
GSK-3β	ATGGGAAAATCAAAAGAAATCAGCC	CGCACCAAAGTACGTTCATCTCTA
Wnt11	AATCAGACGCAACACTGTAAAC	CTCGATGGAGGAGCAGTTC

Primers were provided by Shanghai Sangon Biotechnology (Shanghai, China)

### Western blotting (WB)

Protein was extracted from the harvested FLS of each group. After quantification by BCA Protein Assay Kit (P0010S, Beyotime, Shanghai, China), adding the sample buffer (P0015L, Beyotime, Shanghai, China) to the protein, the protein is separated using SDS-PAGE and transferred to PVDF membrane for further analysis. The amount of protein in each channel was consistent. The solution was sealed with 5% skim milk at room temperature, the primary antibody diluted with Western primary antibody diluent (P0023A, Beyotime, Shanghai, China) was incubated at 4 °C overnight, and then the secondary antibody diluted with sealing solution was incubated at room temperature for 1 h. Detection of protein bands is done using ECL kit (Biosharp, Hefei, China) and analysis using ImageJ software. Antibodies used in this work, including fibronectin (ab268020, 1:1000), β-catenin (ab32572, 1:5000), CCND1 (ab134175, 1:10000), c-Myc (ab32072, 1:1000), and β-actin (ab8226, 1:1000), were all purchased from Abcam Company (Waltham, MA, USA).

### Immunofluorescence

FLS were fixed in 4% paraformaldehyde and washed with PBS. At room temperature, the FLS were infiltrated with 0.1% Triton X-100 and sealed with 1% BSA before being treated with primary antibodies and incubated with the appropriate secondary antibodies. The sample nuclei were stained with DAPI in dark. The fluorescence quenching agent was sealed after PBS washing. Images are obtained under a fluorescence microscope. Antibodies used in this work, including MMP3 (ab52915, 1/250) and Wnt11 (ab31962, 1/200), were all purchased from Abcam Company (Waltham, MA, USA).

### Construction of Wnt11 overexpression vector

#### Adenovirus dilution

After taking out the virus ice bath and thawing, the virus was mixed and packaged with PBS or serum-free medium (containing serum or double antibodies did not affect viral infection) for culture of target cells and stored at 4 °C (for use within 1 week). If the titer of the original virus marker is 1 × 10^10^ PFU/mL, take 10~90 μL conventional medium to obtain the virus with a titer of 1 × 10^9^ PFU/mL [21].

#### Adenovirus infects target cells

- Day 1: Cell preparation—after digestion and counting, the target cells in good growth state were diluted to 3 × 10^5^/mL, and 500 μL/well (1.5×105 cells) was added with 24-well plates. It was incubated overnight in a 5% CO2 incubator at 37 °C.
- Day 2: Viral infection (1/2 small volume infection method) and fluid exchange—add 1/2 volume of fresh culture solution into the 24-well plate and slowly add melted virus for 4 h to make the culture volume reach 500 μL. MOI of 10, 30, 100, 300, and 500 were selected for pretest to find the optimal MOI. Six to 8 h after infection, the culture medium containing virus was aspirated, replaced with fresh complete culture medium, and continued to culture at 37 °C.
- Days 3–4: Fluorescence was observed 36–48 h after infection.

Refer to supplementary material 1 for the specific process of adenovirus packaging.

### Statistical analysis

SPSS 26.0 was used for statistical analysis in this work, with *t*-tests used for data analysis between two groups and analysis of variance used for comparison between three or more groups. Data were expressed as mean ± standard deviation, and *p* < 0.05 was considered significant.

## Results

### WFR alleviates arthritis in CIA rats

**Fig. 1:**
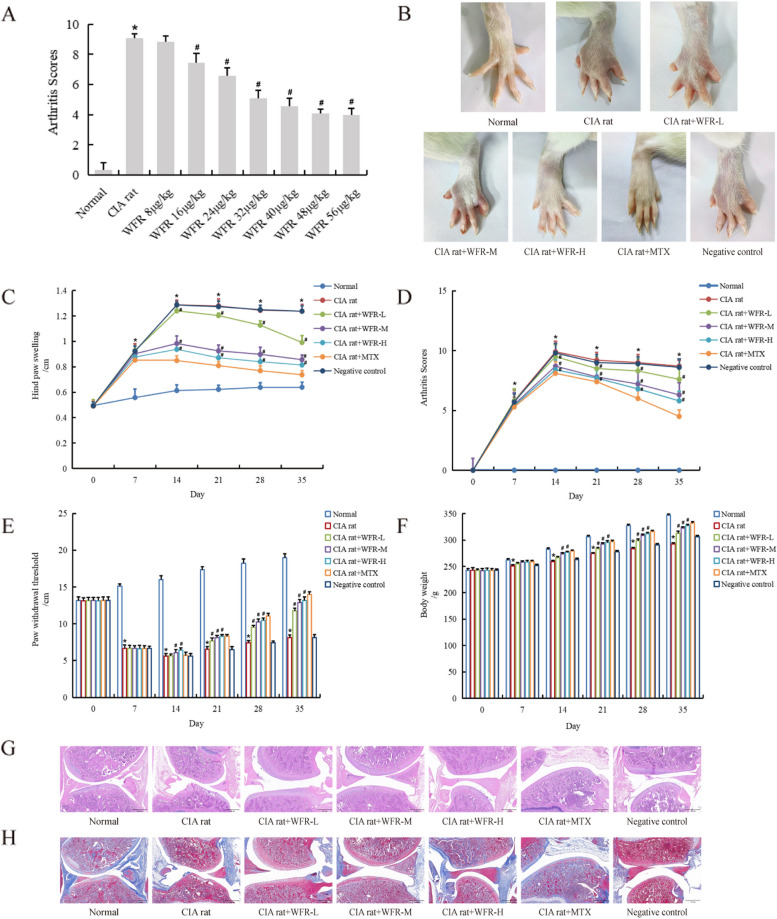
WFR alleviates arthritis in CIA rats. Rats were administered on the 7th day after their first immunization, and arthritis scores were performed on the 35th days. A total of 40 μg/kg, 48 μg/kg, and 56 μg/kg of WFR had good effects on reducing arthritis score, which were determined as the experimental dose (**A**). WFR could significantly reduce paw swelling (**B** and **C**), reduce the arthritis score of CIA rats (**D**), increase the threshold of paw pain withdrawal (**E**), and restore the body weight of CIA rats (**F**). HE staining showed that WFR alleviated synovial hyperplasia and pannus formation in CIA rats (**G**). Masson staining showed that WFR could reduce bone tissue damage in CIA rats (**H**). **C**, **D**, **E**, **F** *CIA group vs normal, #CIA+WFR vs CIA group, *n* = 10. *p < 0.05

We evaluated the effects of various doses of WFR using the RA model rat arthritis score. Rats were administered on the 7th day after their first immunization, and arthritis scores were performed on the 35th day; WFR dosage was selected according to the CIA rat arthritis score, the treatment effects of WFR 40 μg/kg, 48 μg/kg, and 56 μg/kg were better, and there was no significant difference between the 48 μg/kg group and 56 μg/kg group. Therefore, we set WFR 40 μg/kg, 48 μg/kg, and 56 μg/kg as low dose, medium dose, and high dose for subsequent experiments (Fig. 1A).

We evaluated the therapeutic effect of WFR on CIA rats by different RA indexes. The arthritis symptoms of CIA rats were gradually aggravated, including joint inflammation, congestion, inability to bear weight, limited movement, and redness of feet and paws (Fig. 1B). Increases in hind paw swelling (Fig. 1C) and arthritis score (Fig. 1D) were significantly higher in the CIA rats than in the health rats. After 1 week of administration from WFR intragastric administration, the above two indexes in three WFR groups (present dose dependent) and MTX group were significantly decreased (*p* < 0.05). The pain sensitivity of CIA rats could be determined by paw withdrawal threshold (Fig. 1E), and the health status was assessed by measuring the changes in body weight of rats (Fig. 1F). The paw withdrawal threshold and body mass index in WFR (present dose dependent) and MTX groups were significantly higher than those in model group (*p* < 0.05).

HE staining showed that the synovial tissue structure of normal knee joint was clear without hyperplasia. The surface of the joint was smooth, and the articular cartilage and subchondral bone structure were complete. In the CIA model group, synovial tissue was hyperplasia, articular surface was uneven, and cartilage and subchondral bone were extensively destroyed. Compared with model group, WFR alleviated synovial hyperplasia and pannus formation in CIA rats (Fig. 1G). Masson staining showed that the articular cartilage, which contains collagen fibers and ostein, was stained blue, while the bone trabecula in the bone marrow cavity was stained red. In the normal group, the collagen layer of cartilage was blue, while in the CIA rat group, the blue was seriously lost by Masson staining, and the cartilage was destroyed. Some of the cartilage in the drug treatment group remained blue (Fig. 1H). The above results indicate that WFR, like MTX, can significantly reduce bone tissue damage in CIA rats, and WFR is a potential anti-RA monomer compound.

### WFR inhibits the pathological-related genes and the FLS proliferation

**Fig. 2:**
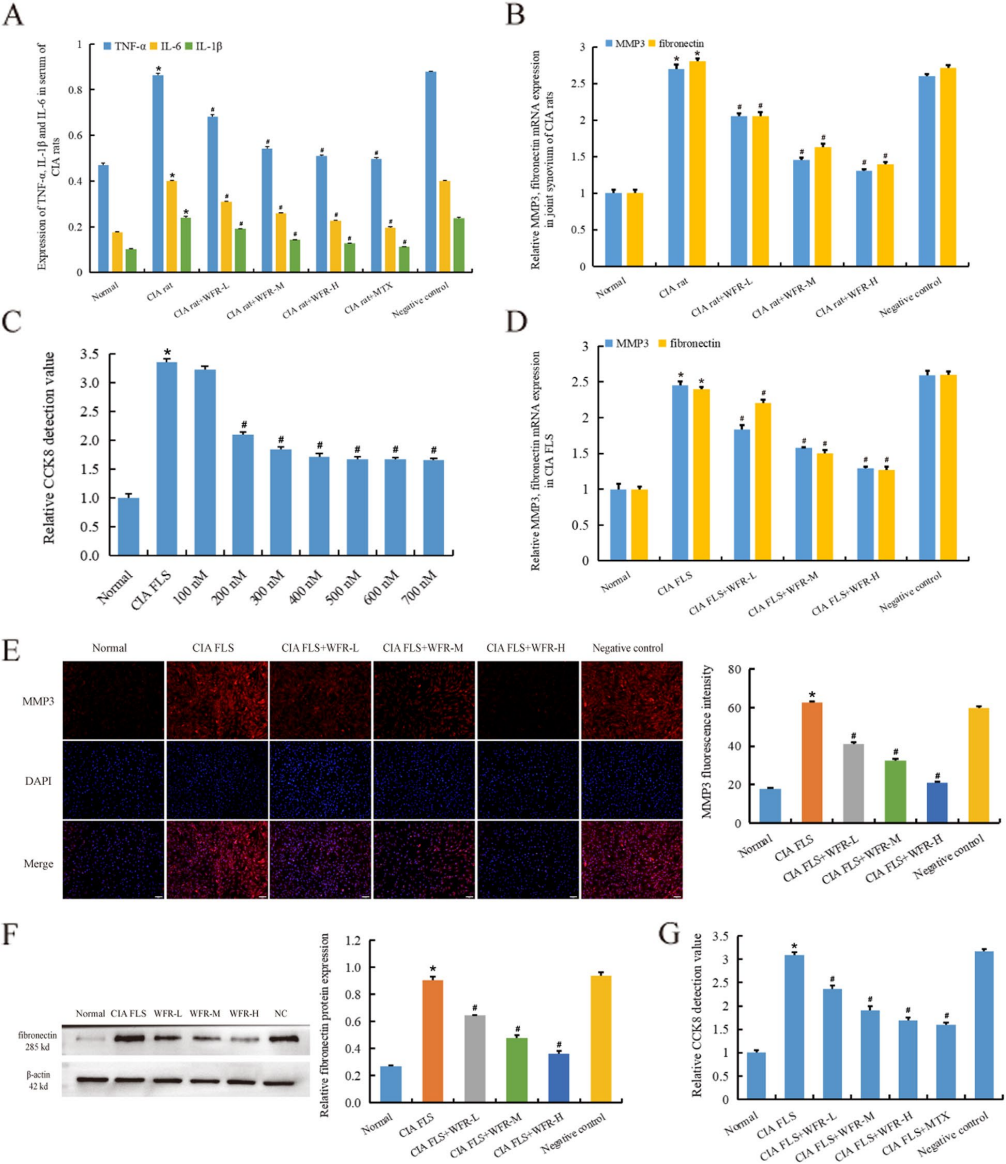
WFR inhibits the pathological-related genes and the FLS proliferation. ELISA showed that WFR effectively reduced TNF-α, IL-6, and IL-1β in serum of CIA rats (**A**). WFR effectively decreased the expression of MMP3 and fibronectin mRNA in the synovium of CIA rats (**B**). The cell dose of WFR was selected according to CCK8 experiment, and WFR significantly inhibited the proliferation of CIA FLS when the dose concentration was 200 nM (**C**). WFR effectively decreased the expression of MMP3 and fibronectin mRNA in CIA FLS (**D**). Immunofluorescence detection showed that WFR decreased the protein expression of MMP3 in CIA FLS (**E**) (scale bar = 50 μm). WB detection showed that WFR decreased the protein expression of fibronectin in CIA FLS (**F**). CCK-8 detection showed that WFR significantly inhibited the proliferation of CIA FLS (**G**). *CIA group vs normal, #CIA group+WFR vs CIA group, *n* = 3. *p < 0.01

ELISA detection showed that three doses of WFR can alleviate arthritis in CIA rats in a dose-dependent manner by downregulating abnormally elevated levels of IL-1β, IL-6, and TNF-α (*p* < 0.01) (Fig. 2A). In joint synovium of CIA rats, WFR significantly reduced abnormally elevated mRNA levels of MMP3 and fibronectin (*p* < 0.01) (Fig. 2B). The three doses of WFR cell assay were determined to be 200 nM (low dose), 300 nM (medium dose), and 400 nM (high dose) by screening cell dosing by CCK8 (Fig. 2C). In CIA FLS, WFR significantly reduced abnormally elevated MMP3 and fibronectin mRNA levels (Fig 2D). Furthermore, immunofluorescence detection showed that WFR decreased the protein expression of MMP3 in CIA FLS (Fig. 2E), and WB detection showed that WFR decreased the protein expression of fibronectin in CIA FLS (*p* < 0.01) (Fig. 2F). CCK-8 detection showed that WFR inhibited the proliferation of CIA FLS, further indicating that WFR can effectively inhibit synovial hyperplasia and alleviate RA (Fig. 2G) (*p* < 0.01)

### Wnt11 significantly higher expression in RA

**Fig. 3:**
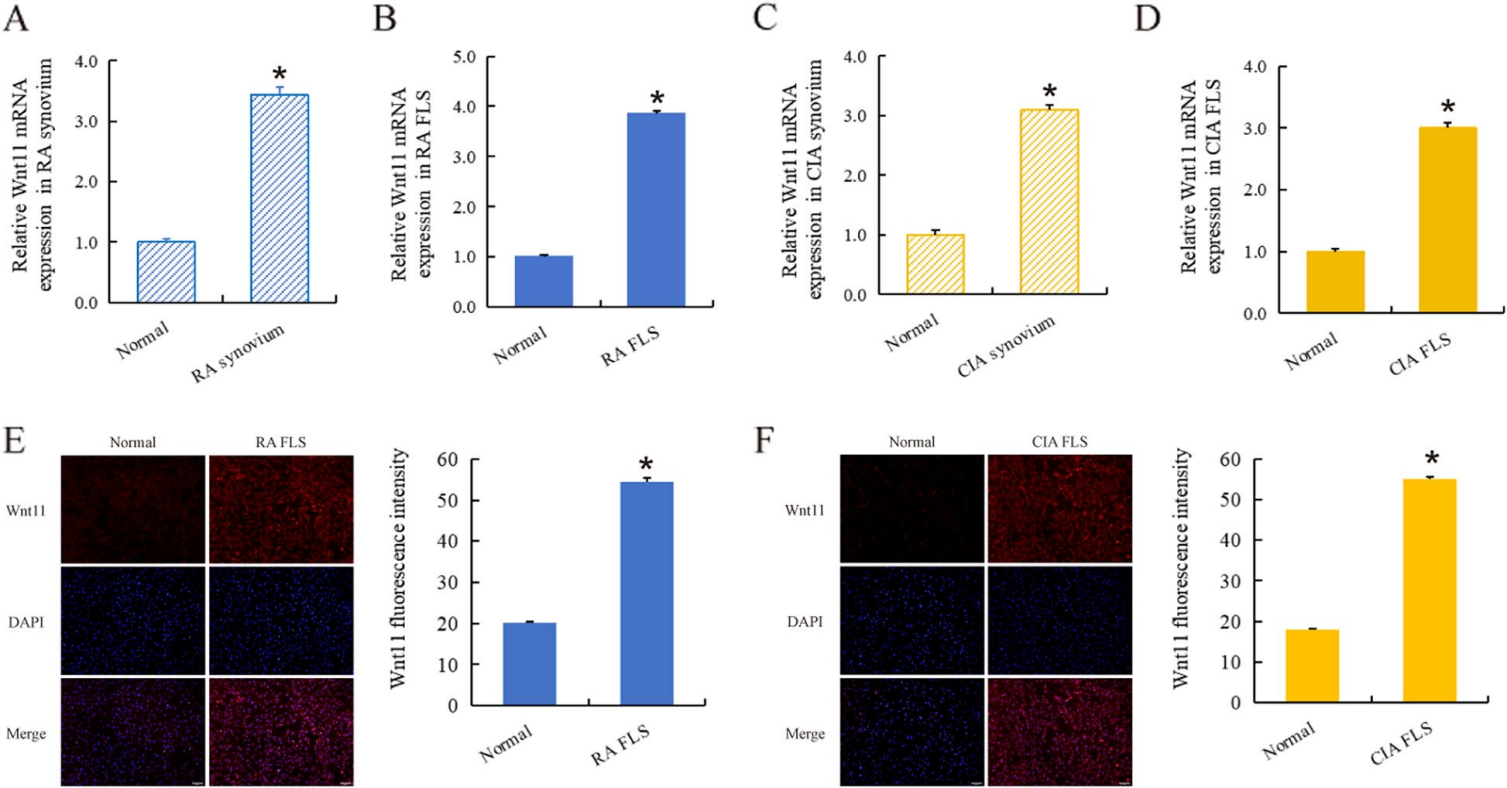
Wnt11 was significantly higher expression in RA. The expression of Wnt11 was significantly increased in RA synovium (**A**), RA FLS (**B**), CIA synovium (**C**), and CIA FLS (**D**), with its expression level about 2–3 times that of the normal group. Immunofluorescence results showed that Wnt11 protein was highly expressed in RA FLS (**E**) (scale bar = 50 μm) and CIA FLS (**F**) (scale bar = 50 μm). *RA/CIA group vs normal, *n* = 3. *p < 0.01

Wnt11 has an important promoting effect on cardiac tissue proliferation and tumor cell proliferation [15–19]. Given the similarity between synovial hyperplasia and angiogenesis in RA and cardiac tissue hyperplasia and tumor pathology, we investigated the expression changes and roles of Wnt11 in RA. We found for the first time that the expression of Wnt11 was significantly increased in RA synovium (Fig. 3A), RA FLS (Fig. 3B), CIA synovium (Fig. 3C), and CIA FLS (Fig. 3D), with its expression level about 2–3 times that of the normal group, indicating that Wnt11 was involved in RA pathology. Furthermore, immunofluorescence detection showed that Wnt11 protein was highly expressed in RA FLS (Fig. 3E) and CIA FLS (Fig. 3F) (*p* < 0.01).

### WFR inhibits abnormally elevated Wnt11 expression in RA

**Fig. 4:**
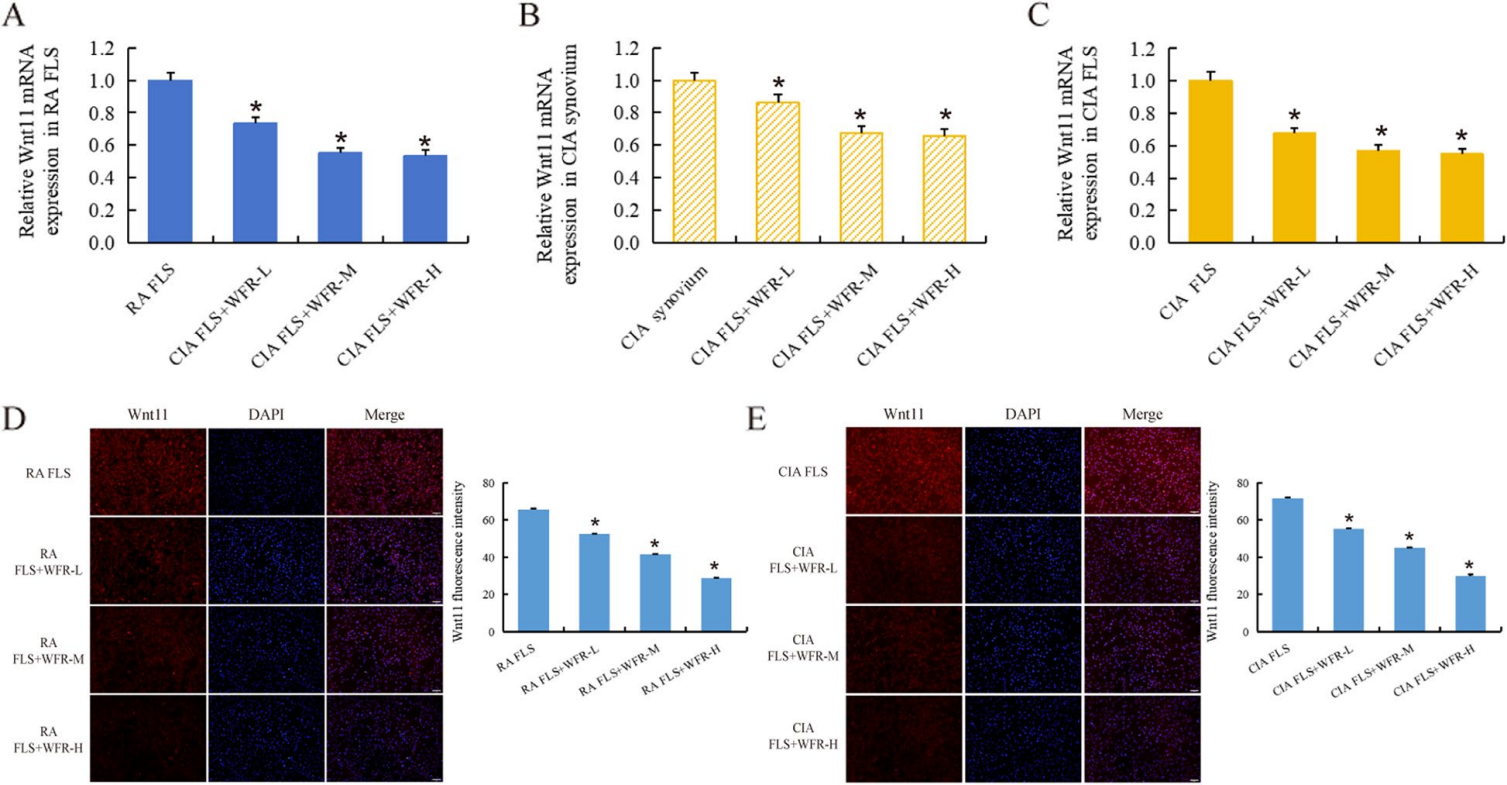
WFR inhibits abnormally elevated Wnt11 expression in RA. RT-qPCR results showed that WFR reduced mRNA levels of abnormally elevated Wnt11 in RA FLS (**A**), CIA rat synovium (**B**), and CIA FLS (**C**). Immunofluorescence results showed that WFR significantly reduced the amount of Wnt11 protein in RA FLS (**D**) (scale bar = 50 μm) and CIA FLS (**E**) (scale bar = 50 μm). *RA/CIA group vs normal, #RA/CIA group+WFR vs RA/CIA group, *n* = 3. *p < 0.01

RT-qPCR results showed that WFR reduced mRNA levels of abnormally elevated Wnt11 in RA FLS (Fig. 4A), CIA rat synovium (Fig. 4B), and CIA FLS (Fig. 4C) (*p* < 0.01). Immunofluorescence results showed that WFR significantly reduced the amount of Wnt11 protein in RA FLS (Fig. 4D) and CIA FLS (Fig. 4E) (*p* < 0.01).

### WFR inhibits the activation of Wnt/β-catenin signaling pathway

**Fig. 5:**
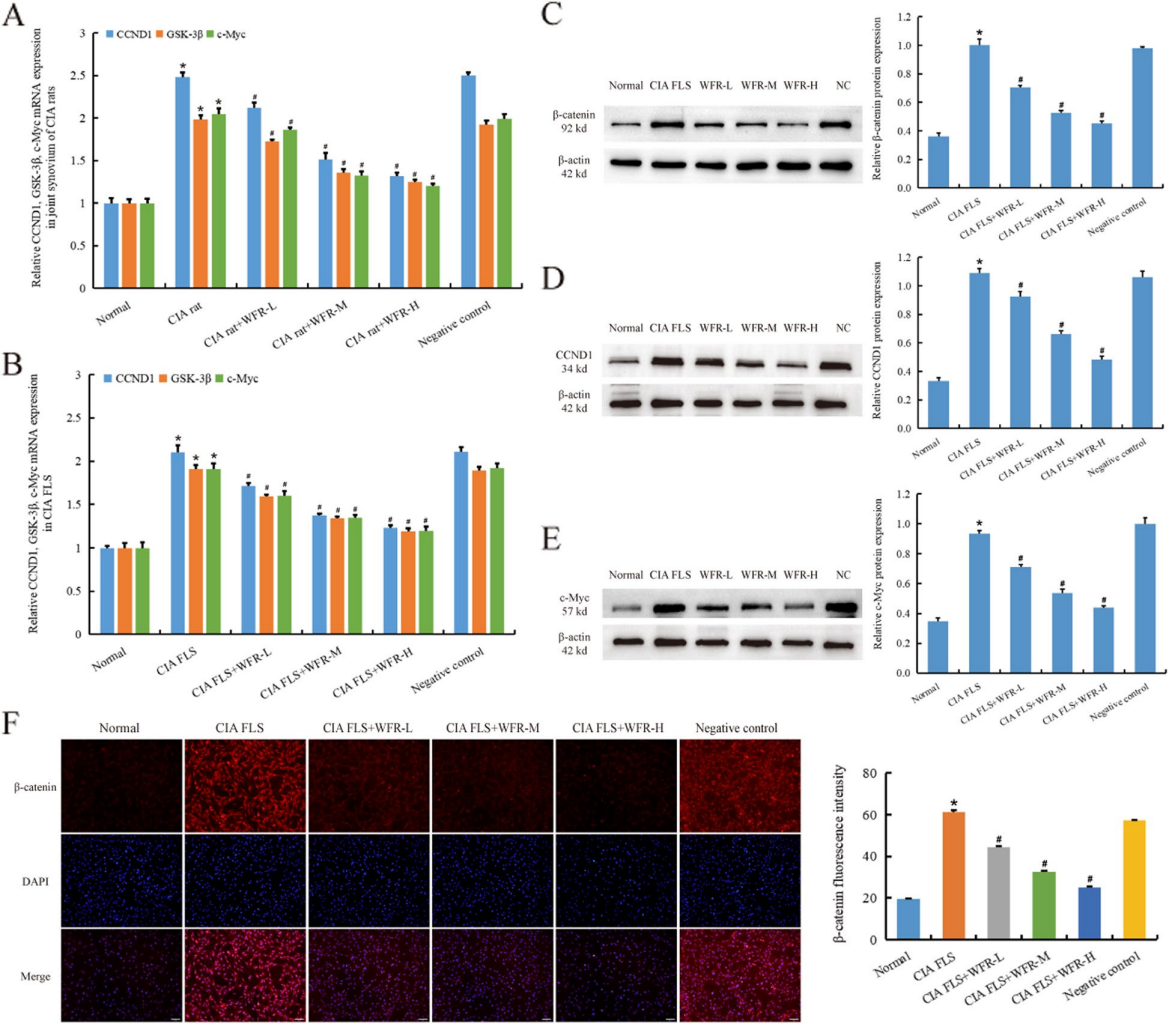
WFR inhibits the activation of Wnt/β-catenin signaling pathway. RT-qPCR results showed that WFR effectively decreased the expression of CCND1, GSK-3β, and c-Myc mRNA in the joint synovium of CIA rats (**A**), WFR effectively decreased the expression of CCND1, GSK-3β, and c-Myc mRNA in CIA FLS (**B**). WB detection showed that WFR decreased the protein expression of β-catenin (**C**), CCND1 (**D**), and c-Myc (**E**) in CIA FLS. Immunofluorescence assay showed that WFR reduced the expression of β-catenin into the nucleus in CIA FLS (**F**) (scale bar = 50 μm). *CIA group vs normal, #CIA group+WFR vs CIA group, *n* = 3. *p < 0.01

We detected whether WFR inhibited FLS activation through the Wnt/β-catenin signaling pathway *in vivo* and *in vitro* experiments. In CIA rat synovium (Fig. 4A) and CIA FLS (Fig. 4B), WFR significantly reduced the mRNA levels of CCND1, GSK-3β, and c-Myc (*p* < 0.01). According to WB data, WFR reduced the protein expression of β-catenin (Fig. 4C), CCND1 (Fig. 4D), and c-Myc (Fig. 4E) in CIA FLS (*p* < 0.01). Immunofluorescence recorded the entry of β-catenin into the nucleus, and WFR significantly reduced the amount of β-catenin protein in the CIA FLS nucleus (Fig. 4F) (*p* < 0.01) Fig 5.

### Overexpression of Wnt11 reverses the effect of WFR

**Fig. 6:**
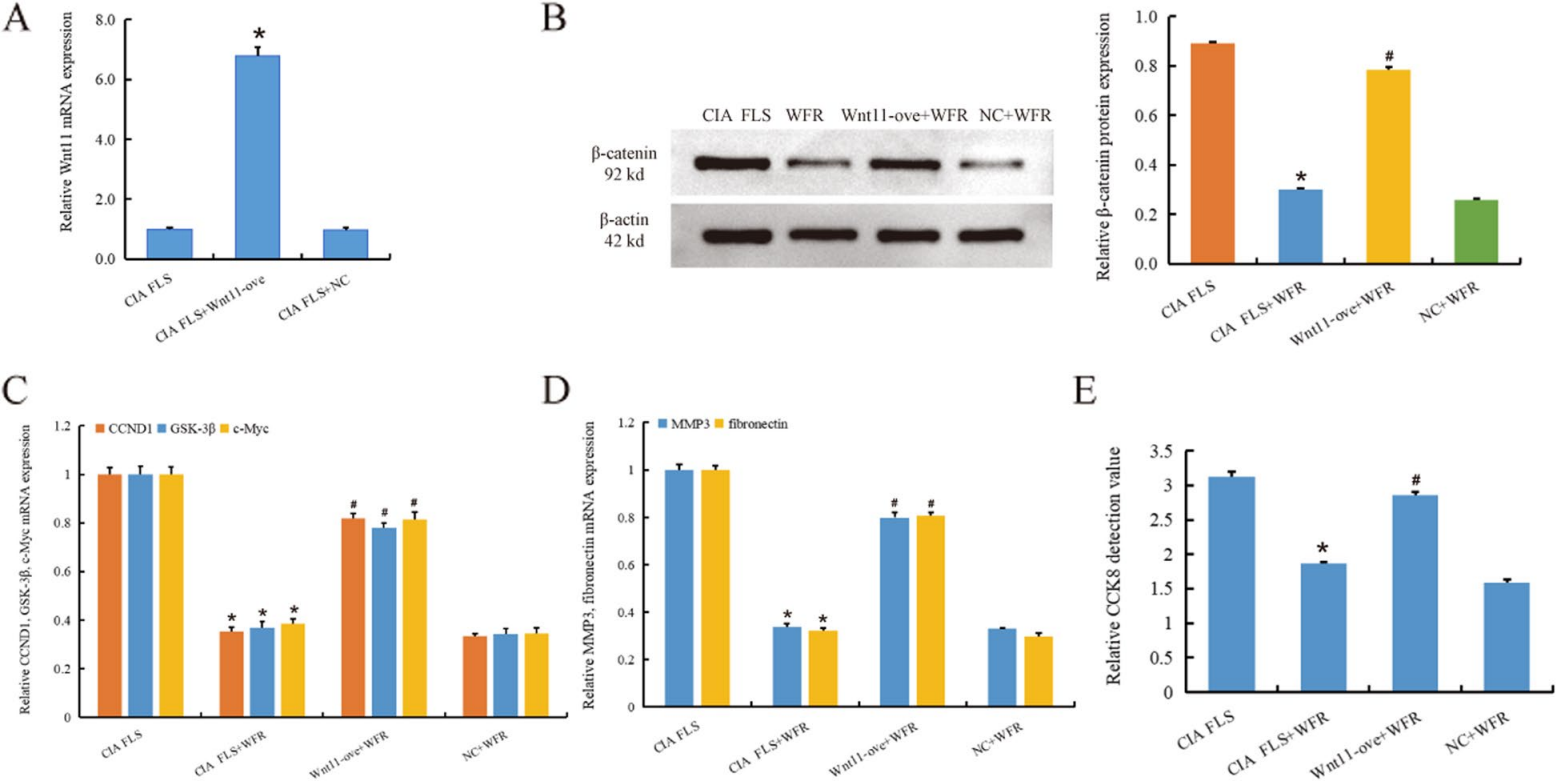
Overexpression of Wnt11 reversed the effect of WFR. RT-qPCR results showed that Wnt11 mRNA expression significantly increased after Wnt11 overexpression (Wnt11-ove) (**A**). WB results showed that Wnt11-ove reversed the inhibitory effect of WFR on β-catenin protein expression in CIA FLS (**B**). RT-qPCR results showed that Wnt11-ove interfered with WFR to inhibit the mRNA expression of CCND1, GSK-3β, and c-Myc in CIA FLS (**C**). Wnt11-ove interfered with WFR inhibition of MMP3 and fibronectin mRNA expression in CIA FLS (**D**). CCK-8 results showed that Wnt11-ove interfered with the inhibitory effect of WFR on RA FLS (**E**). For **A**, *CIA FLS+Wnt11 ove vs CIA FLS. For **B**, **C**, **D**, **E**, *CIA FLS+WFR vs CIA FLS, #Wnt11 ove+WFR vs CIA FLS+WFR, *n* = 3. *p < 0.01

RT-qPCR results showed that Wnt11 mRNA expression significantly increased after Wnt11 overexpression (Wnt11-ove) (*p* < 0.01) (Fig 6A), indicating that the overexpression was successfully constructed. Wnt11-ove reversed the inhibitory effect of WFR on Wnt/β-catenin signaling pathway. The expression level of β-catenin protein in the nucleus was considerably reduced following the addition of WFR to CIA FLS (*p* < 0.01). However, the expression of β-catenin protein in Wnt11-ove+WFR group was higher than that in WFR group (Fig. 6B). RT-qPCR results showed that WFR suppressed the expression of CCND1, GSK-3β, and c-Myc in CIA FLS, while Wnt11-ove interfered with the action of WFR (*p* < 0.01) (Fig. 6C). In addition, WFR inhibited the expression of pathological genes (MMP3 and fibronectin) in RA FLS, while Wnt11-ove interfered with this effect (*p* < 0.01) (Fig. 6D). CCK-8 detection also indicated that Wnt11-ove interfered with the inhibitory effect of WFR on RA FLS (*p* < 0.01) (Fig. 6E). These results further confirm that WFR inhibits the activation of Wnt11/β-catenin signaling pathway, thereby inhibiting RA pathology.

## Discussion

Due to the incomplete understanding of the pathophysiology of RA, it is challenging to achieve targeted therapy for RA [22]. Although great progress has been made in improving antirheumatic drugs for RA in recent years, it is still necessary to understand the development and molecular mechanism of RA clinically. FLS are the main effector of synovial tissue proliferation and inflammation in RA, and FLS-mediated inflammatory response and pannus formation are important pathological processes in RA formation [23]. TCM has become a research hotspot in the treatment of RA due to its multi-component, multi-target, multi-pathway, and only mild side effects. From the perspective of FLS, exploring the mechanism of WFR in the treatment of RA will provide a novel perspective for TCM to inhibit the proliferation of FLS and the secretion of inflammatory mediators, induce the apoptosis of FLS, and thus treat RA.

The pathological characteristics of CIA rats are similar to those of human RA, which are widely used to test RA treatment drugs and RA pathological research [24]. After WFR treatment, the degree of hind paw swelling, histopathological injury, and inflammation in CIA rats is significantly reduced. An important factor in the etiology of RA is the dysregulation of the cytokine network [25]. In our work, both WFR and MTX drastically downregulated the levels of IL-6, IL-1β, and TNF-α in CIA rats and inhibited the inflammation of RA. In RA, fibronectin is one of the most prevalent proteins [26], and MMP3 is a common marker of disease activity, forecasting of disease outcome, and therapy response [14]. WFR downregulated the mRNA and protein expression of MMP3 and fibronectin in the synovium of CIA rats and effectively alleviated the disease activity of RA. In addition, WFR inhibited FLS proliferation and improved RA in a dose-dependent manner. At the cellular level, we demonstrated that serum containing WFR inhibited the proliferation of FLS, showing that WFR had a beneficial effect on the synovial pathological alterations associated with RA.

Wnt11 is a secreted glycoprotein associated with the extracellular matrix in many tissues [27]. Wnt11, a new gene associated with early onset osteoporosis and the main Wnt ligand related to bone fragility and EOOP, which mutations in this gene were described to be related to autosomal dominant osteoporosis, is required for osteoblastogenesis [27]. During glucocorticoid-induced osteogenesis, Wnt11 expression increased [28]. Like other atypical Wnts, Wnt11 acts through a calcium-dependent pathway. Conversely, there is evidence that Wnt11 also induces osteoblast differentiation through typical signaling [29]. Wnt11 may be a potential target for controlling the course of RA disease based on its multimode effect on the genesis of the disease.

We found that the expression of Wnt11 was significantly increased in RA, and WFR could significantly inhibit the abnormally high expression of Wnt11. Further studies showed that WFR also acted on the expression of Wnt/β-catenin-related proteins. WFR inhibited the abnormally high expression of β-catenin, CCND1, GSK3β, and c-Myc in CIA FLS. Furthermore, according to immunofluorescence results, WFR reduced β-catenin entrance into the nucleus, further supporting that WFR acted via the Wnt/β-catenin signaling pathway.

In the process of RA disease development, not only the Wnt11 signaling pathway is involved in the regulation of the disease progression but also other pathways are also widely involved in various processes of the disease, such as NF-κB, JNK, and MAPK, and different signaling pathways interact with each other. The relationship between Wnt11 and these pathways is not fully elucidated. Therefore, through in-depth study of the role of various factors in the Wnt11 pathway and exploration of the relationship with other pathways, new ideas and directions will be provided for the pathogenesis and treatment of RA.

## Conclusions

This study found that WFR inhibited the activation of the Wnt11/β-catenin signaling pathway, thereby preventing the development of RA FLS and alleviating RA symptoms. Wnt11 was a direct target of WFR. This study provides a new molecular mechanism for WFR to improve RA, which is helpful to promote the clinical development of WFR.

### Supplementary Information

**Additional file 1. **Figure 2F Fibronectin (abcam: ab268020). β-actin (abcam: ab8226). Figure 5C β-catenin (abcam: ab32572). β-actin (abcam: ab8226). Figure 5D Cyclin D1 (abcam: ab134175). β-actin (abcam: ab8226). Figure 5E c-Myc (abcam: ab32072). β-actin (abcam: ab8226). Figure 6B β-catenin (abcam: ab32572). β-actin (abcam: ab8226).

**Additional file 2. **Supplementary material 1. Adenovirus operation (Hanheng, Shanghai, China).

## Data Availability

Not applicable

## Abbreviations

CCND1: Cyclin D1
CIA: Collagen-induced arthritis
c-Myc: Cellular-myelocytomatosis viral oncogene
FLS: Fibroblast-like synoviocytes
GSK-3β: Glycogen synthase kinase-3β
MTX: Methotrexate
MMP3: Matrix metalloproteinase 3
RA: Rheumatoid arthritis
TNF-α: Tumor necrosis factor-α
TCM: Traditional Chinese medicine
TwHF: *Tripterygium wilfordii* Hook. f.
WFR: Wilforine
