# Supplementary material for: Wilforine inhibits rheumatoid arthritis pathology through the Wnt11/β-catenin signaling pathway axis
Source: Arthritis Res Ther. 2023 Dec 14;25:243. doi: 10.1186/s13075-023-03224-2 (PMC10720104; doi:10.1186/s13075-023-03224-2)

Figure 2F

Fibronectin (abcam: ab268020)

β-actin (abcam: ab8226)

Sample 1

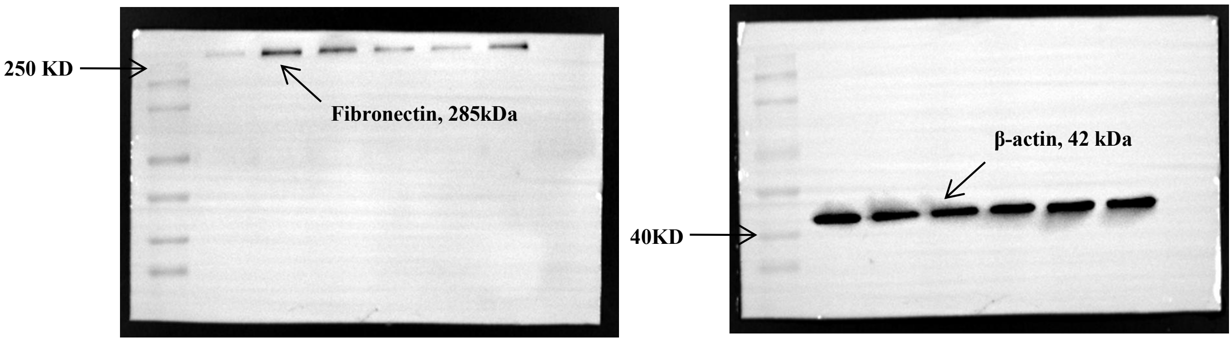

Sample 2

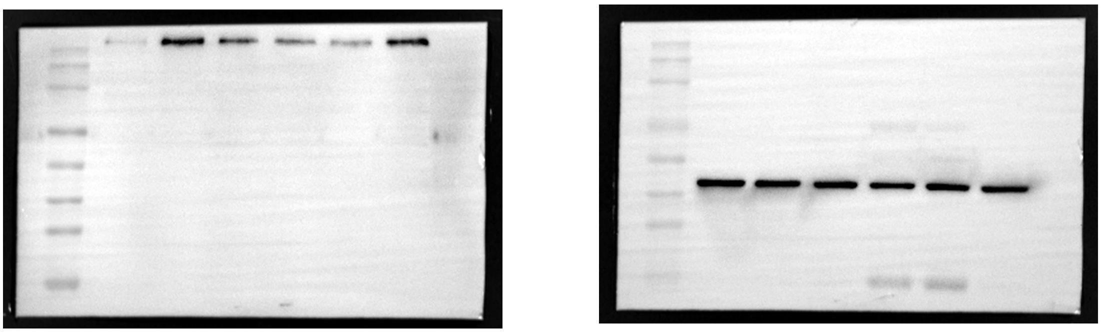

Sample 3

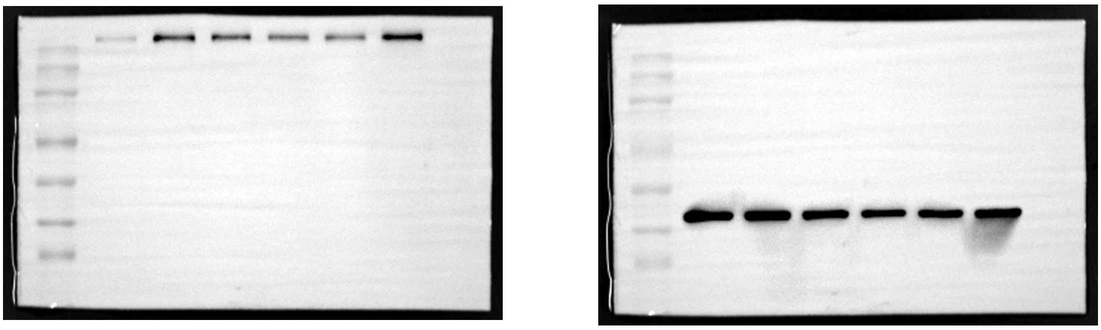

Figure 5C

β-catenin (abcam: ab32572)

β-actin (abcam: ab8226)

Sample 1

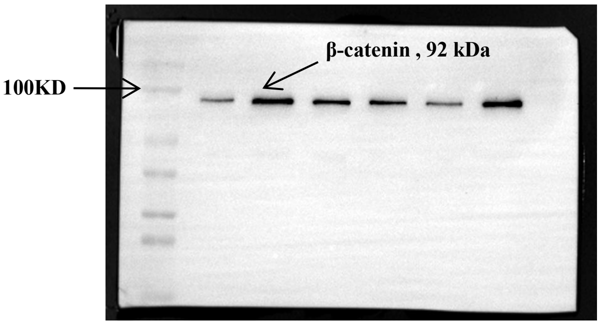

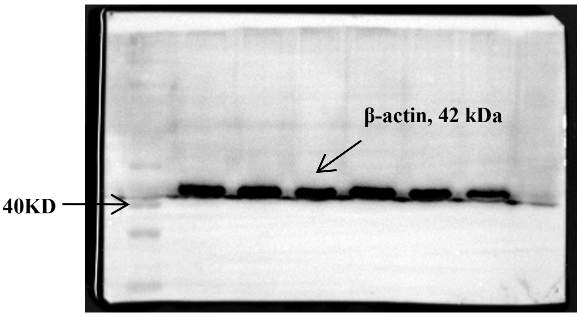

Sample 2

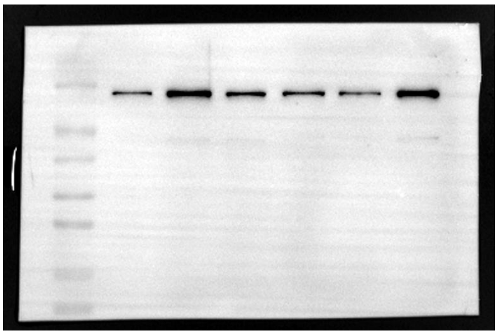

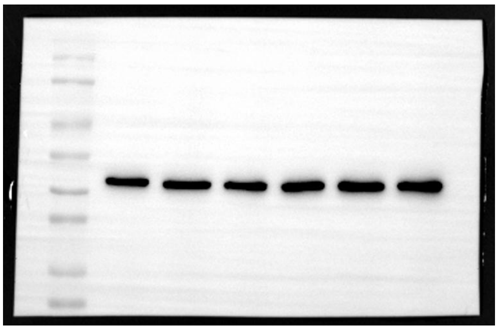

Sample 3

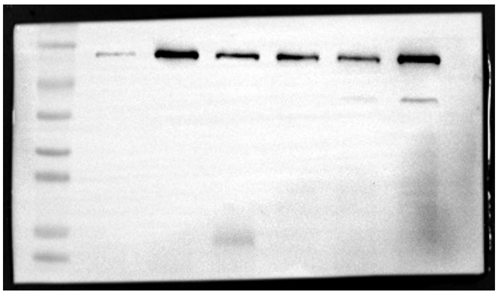

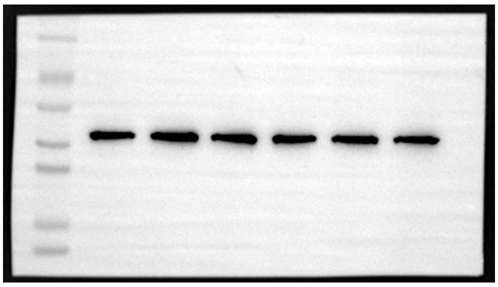

Figure 5D

Cyclin D1 (abcam: ab134175)

β-actin (abcam: ab8226)

Sample 1

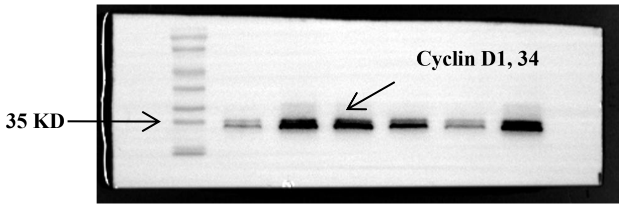

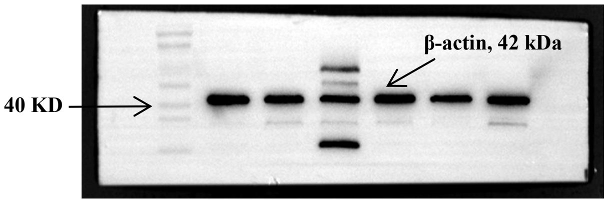

Sample 2

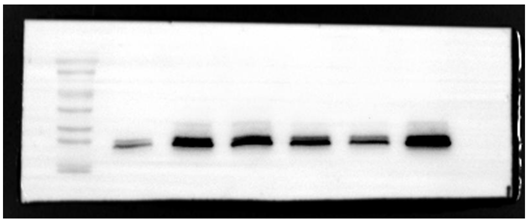

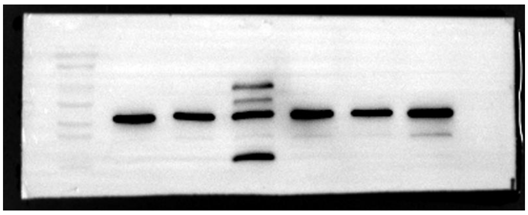

Sample 3

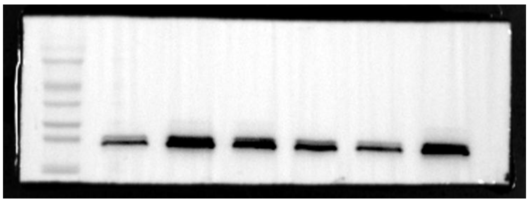

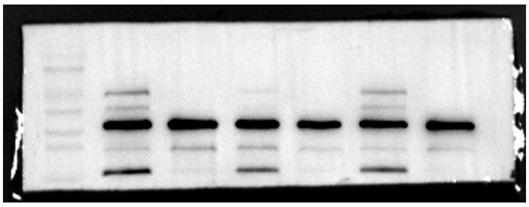

Figure 5E

c-Myc (abcam: ab32072)

β-actin (abcam: ab8226)

Sample 1

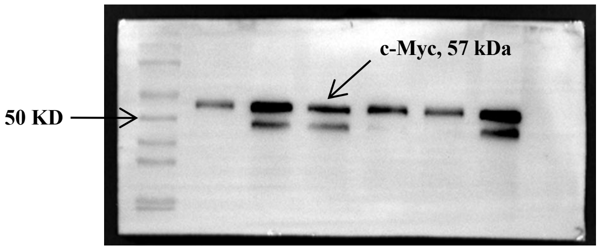

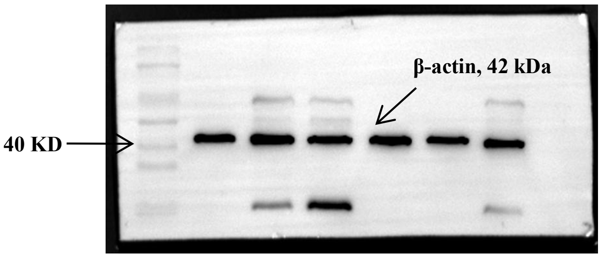

Sample 2

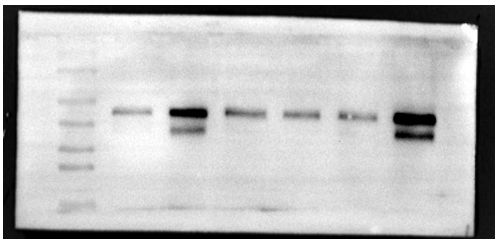

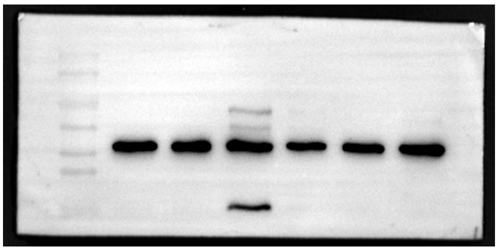

Sample 3

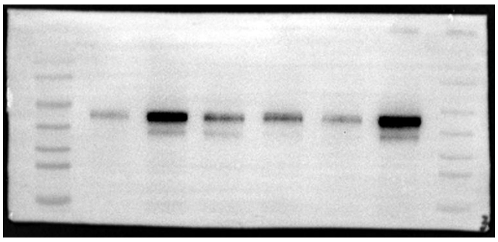

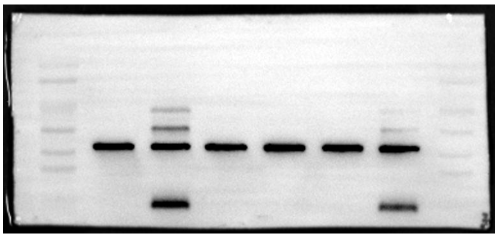

Figure 6B

β-catenin (abcam: ab32572)

β-actin (abcam: ab8226)

Sample 1

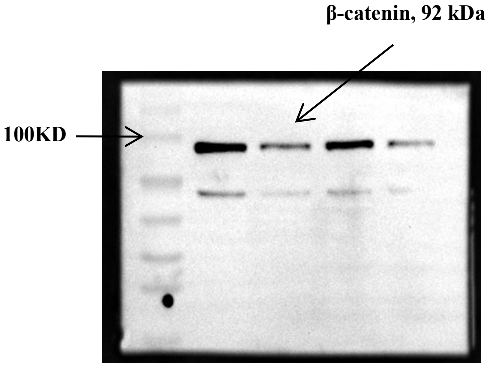

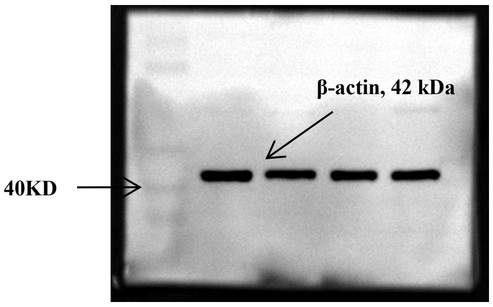

Sample 2

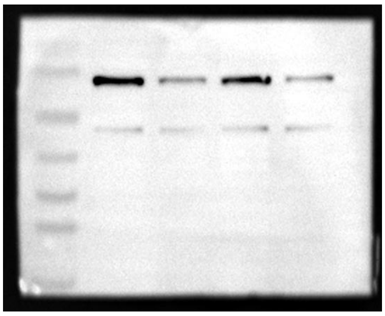

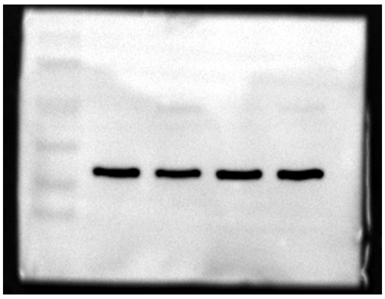

Sample 3

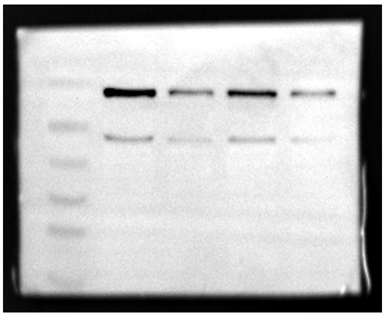

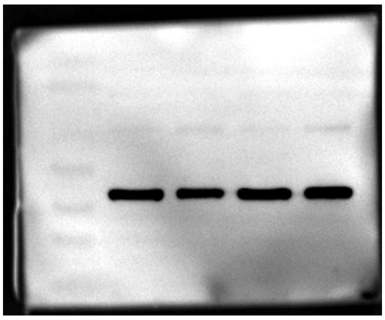

Supplement: Supplementary file 1 — Additional file 1. Figure 2F Fibronectin (abcam: ab268020). β-actin (abcam: ab8226). Figure 5C β-catenin (abcam: ab32572). β-actin (abcam: ab8226). Figure 5D Cyclin D1 (abcam: ab134175). β-actin (abcam: ab8226). Figure 5E c-Myc (abcam: ab32072). β-actin (abcam: ab8226). Figure 6B β-catenin (abcam: ab32572). β-actin (abcam: ab8226). [file 13075_2023_3224_MOESM1_ESM.pdf]
