# Supplementary material for: Wilforine inhibits rheumatoid arthritis pathology through the Wnt11/β-catenin signaling pathway axis
Source: Arthritis Res Ther. 2023 Dec 14;25:243. doi: 10.1186/s13075-023-03224-2 (PMC10720104; doi:10.1186/s13075-023-03224-2)
Supplement: Supplementary file 2 — Additional file 2. Supplementary material 1. Adenovirus operation (Hanheng, Shanghai, China). [file 13075_2023_3224_MOESM2_ESM.docx]

**Supplementary material 1. Adenovirus operation** (Hanheng, Shanghai, China)

1. Dilution of adenovirus

After the virus is taken out and thawed in ice bath, PBS or serum-free medium (containing serum or double antibody does not affect the virus infection) for culturing the target cells is used to mix and pack the virus, and then store it at 4 ℃ (used within one week). If the titer of the original virus marker is 1*10^10^PFU/mL, take 10 μL to 90 μL of conventional culture medium to obtain a virus with a titer of 1*10^9^PFU/mL.

2. Adenovirus infects target cells

MOI groping. MOI (multiplicity of infection) refers to the number of viruses infected by each cell. Generally, the higher the MOI, the higher the expression of the target protein, but the greater the toxicity. For cells with active division, such as HeLa and 293 cells, when MOI = 1 ~ 3, more than 80% of the cells express the target gene. For non dividing cells, such as primary cells, the infection efficiency is low.

(24 hole plate groping MOl)

Day 1: cell preparation

Take 293T cells as an example, after digesting and counting the target cells in good growth state, dilute them to 3*10^5^/mL, add 24 well plates, 500 μL/well (1.5×10^5^ cells). Put it into a 5% CO_2_ incubator at 37 ℃ and incubate overnight.

Day 2: virus infection (1/2 small volume infection method) and fluid exchange

Add 1/2 volume of fresh culture solution to 24 well plate, add slowly melted virus for 4h, and make up to 500 ul culture volume. The MOI of 10, 30, 100, 300 and 500 were selected for pre experiment to find the optimal MOI. 6-8 h after infection, the culture medium containing virus was aspirated and replaced with fresh complete culture medium, and the culture was continued at 37 ℃.

Day 3-4: Observation of fluorescence

Fluorescence was observed at 36-48 h after infection. In the group with infection efficiency of about 80% and good cell growth, the corresponding infection conditions and MOI can be used as the reference MOI for subsequent infection experiments.

3. Adenovirus infection of adherent cells

Day 1: cell preparation

Take 293T cells as an example, after digesting and counting the target cells in good growth state, dilute them to 3*10^5^F/mL, add 24 well plates, 500L/well (1.5×10^5^ cells). Put it into a 5% CO_2_ incubator at 37 ℃ and incubate overnight.

Day 2: virus infection (1/2 small volume infection method) and fluid exchange

In case of virus infection, add 1/2 volume of fresh culture medium, and make up to normal culture volume after adding adenovirus for 4 h.

The specific steps are as follows:

Before infection, take out the virus from the refrigerator and slowly melt it on the ice, suck out the original culture medium of the cells, add 1/2 volume of fresh culture medium, add the appropriate volume of virus according to the MOI value explored, and mix it gently for infection (amount of virus per well (UL) = MOlx cells / virus drop (PFU/mL) × 1000)。 4 h after infection, it was supplemented to the complete culture volume.

Day 3-4: Observation of fluorescence

24-48 h after infection, the GFP expression efficiency of the virus with GFP reporter gene can be observed by fluorescence microscopy.
